# Assessing the Effectiveness of Intergenerational Virtual Service-Learning Intervention on Loneliness and Ageism: A Pre-Post Study

**DOI:** 10.3390/healthcare10050893

**Published:** 2022-05-12

**Authors:** Zo Ramamonjiarivelo, Randall Osborne, Oren Renick, Keya Sen

**Affiliations:** 1 School of Health Administration, College of Health Professions, Texas State University, San Marcos, TX 78666, USA; renick@txstate.edu (O.R.); keyasen@txstate.edu (K.S.); 2 Department of Psychology, College of Liberal Arts, Texas State University, San Marcos, TX 78666, USA; ro10@txstate.edu

**Keywords:** service-learning, loneliness, ageism

## Abstract

Background: Service-learning is an effective intervention to solve social issues. The purpose of this study is to assess the effectiveness of intergenerational virtual service-learning on loneliness and ageism. Method: This study used a pre-post design. A group of undergraduate students were randomly assigned to a “service-learning” project (*n* = 18). They were paired with seniors (*n* = 22) to have at least a 30-min weekly virtual interaction for six weeks. The following scales were used: the Aging Semantic Differential (ASD) Scale, the UCLA Loneliness Scale, a one-item researcher generated Likert-rating of loneliness, and two-item researcher generated Likert-rating of student competence. Results: Among college students, the service-learning group showed lower ASD and ageism scores at the post-test compared to the non-service-learning group, t (1, 40) = −2.027, *p* = 0.049; t (1, 40) = −2.102, *p* = 0.042, respectively. Among seniors, loneliness scores on the UCLA Scale and the one-item scale of loneliness dropped significantly from pre- to post-interactions with students, t (1, 19) = 2.301, *p* = 0.033, and t (1, 22) = 2.412, *p* = 0.009, respectively. Conclusion: Virtual service-learning is an effective way to solve social issues such as loneliness and ageism.

## 1. Introduction

For at least a generation, advocates for the improved quality of life in older adults have identified social well-being as a crucial aspect of an individual’s overall health. Calling for a culture change in societal attitudes about the elderly and their care, these transition figures have focused on the plagues of the elderly—helplessness, boredom, and loneliness. Helplessness is felt when one always receives care but can never give care. Boredom results when one’s life has no variety or spontaneity. Loneliness is the pain of wanting but not having companionship [1]. The authors consider the potential of a program of intergenerational service-learning to lessen the impact of the plagues on the elderly, concentrating specifically on the plague of loneliness, while reducing ageism by college students.

Service-learning is an experiential learning. Bringle and Hatcher [2] define service-learning as a “course-based, credit-bearing educational experience in which students (a) participate in an organized service activity that meets identified community needs and (b) reflect on the service activity in such a way as to gain further understanding of a course content, a broader appreciation of the discipline, and an enhanced sense of civic responsibility” [2]. As such, service-learning is a means to educate students about social/community problems by involving students in community-based services/activities. Another learning component of service-learning consists of students writing a diary of their daily activity and a reflection on what they have learned from the activities [2]. While educating students about these social/community issues, they are also part of the solutions to these issues. Intergenerational service-learning is an extension of service-learning where college students are engaged in activities that involve individuals of older generation.

Humans are social beings who thrive from having relationships with others. Loneliness, described as “the subjective perception of lack of meaningful relationship” and “social isolation” portrayed as an objective evaluation of scarce social engagements and social contacts have been concerning social issues declared as a “global epidemic” [3], page 456, among the world’s elderly population by the US Surgeon General Vivek Murthy in 2017 [4]. In fact, about 24% of non-institutionalized older adults have experienced social isolation and 35% of adults ≥45 years old have felt lonely [5]. Many older adults without a strong network of friends or family are at a disadvantage with shrunken social network with very limited scope of interaction [6].

Both loneliness and social isolation may be associated with negative health outcomes. For instance, the most recent report from the National Academies showed that individuals who are socially isolated are more likely to die younger and develop dementia compared with those who are not socially isolated. Individuals who experience poor social relationships, described as social isolation or loneliness, have 29% higher risk of coronary heart disease and 39% higher risk of stroke compared with those who have rich social relationships [5].

In addition, the risk of death among lonely individuals who have heart failure is four times higher than those with heart failure but are not lonely. Moreover, lonely individuals have 68% higher risk of hospitalization and 57 % higher risk of emergency department visits compared with those who are not lonely [5], as well as increased risk of depression, alcoholism, sleep problems, change in immunological system, Alzheimer’s disease, poor general health status, decreased wisdom, anxiety, suicide, accelerated cognitive decline, hypertension, diabetes, and death [7,8,9,10,11,12,13].

Older individuals tend to experience loneliness or social isolations more than younger individuals because they tend to live alone and their poorer health and weaker physical conditions may limit their ability to have some meaningful social interactions [3]. In fact, many older adults without a strong network of friends or family are at a disadvantage with shrunken social network with very limited scope of interaction [6]. In addition, the older generation might not be as comfortable speaking to someone 50 years younger in comparison to someone of the same age; however, this does not mean that the older adults do not crave that interpersonal connection.

Social interaction through a service-learning program has been found to enhance connectivity, and reduce the risk of losing the motivation to maintain an active and healthy lifestyle [8]. Moreover, intergenerational service-learning conversations foster the comfort level of the youth and that of seniors with time and often make the senior feel that he/she is cared for while in conversation [14]. Additionally, such programs are found to improve 54% of the youth social skills [15].

The majority of service-learning projects have been conducted face-to-face where college students have direct/physical interactions with their assigned community partners. However, due to COVID-19 pandemic that mandated total home confinement for several months, face-to-face service-learning was not an option. Fortunately, advances in communication technologies provide opportunities to conduct service-learning activities virtually, if feasible and appropriate. Regardless, little is known about the effectiveness of virtual service-learning in solving social issues and educating students about these issues.

The purpose of this study is to build upon prior intergenerational service-learning studies by assessing the effectiveness of virtual intergenerational service-learning on loneliness and ageism, among older individuals, as well as ageism, among college students. The World Health Organization defines ageism as “the stereotypes, prejudice, and discrimination toward others or oneself based on age” [16].

### 1.1. Literature Review of Interventions to Reduce Loneliness and Social Isolation

A body of interventions has been undertaken to alleviate the burden of loneliness and social isolation among elder adults both at the national and the organizational/community levels. For instance, at the national level, the National Academies created the Committee on the Health and Medical Dimensions of Social Isolation and Loneliness in Older Adults in 2018. That Committee is responsible for collecting evidence regarding the negative impacts of social isolation and loneliness on quality of life of adults ≥50 years old, as well as recommending strategies, to be implemented by healthcare providers, to reduce the negative health outcomes of social isolation and loneliness [5]. In another instance, the British and Japanese governments appointed a Ministry of Loneliness in 2018 and 2021, respectively, to create nationwide programs to reduce loneliness and its side effects, such as suicide, which has increased during COVID19 pandemic [9,17,18].

In addition, several interventions to tackle loneliness and/or social isolation have been implemented at the community or local level. The scoping review and meta-analysis of Masi et al., (2011) suggests that the aims of such interventions may fall into four major categories: to improve social skills, to grow social support, to offer more opportunities for social interaction, and to deal with maladaptive social cognition [19]. Direct interventions that specifically address social isolation and loneliness, such as a one-on-one interaction between individuals who experience loneliness and social isolations and volunteers who have accepted to interact with the affected individuals has been deemed more effective than interventions that target a group of lonely individuals [5]. An example of one-on one interaction is the Intergenerational Social Support through service-learning that provides individuals a heightened chance of connection with the society [20]. Community driven opportunities for seniors to be socially integrated, can enhance the quality of life [21]. Furthermore, targeted measures through service-learning program interventions such as 30-min storytelling or sharing of daily life experiences in weekly conversations develop understanding of the complex relationships between the aged and the student that might enhance positive emotions [22]. Seniors do not feel that students condescend to them, and this often helps to elevate positive experiences. With time, this increases the feeling in older adults that they are being listened to and not invisible [14].

Since the aging population and loneliness are global issues, intergenerational service-learning has become a globally accepted pedagogy to address both issues [23,24,25]. Intergenerational service-learning is especially advantageous because both the older and younger generations can benefit from it. First, among the older generation, social interaction through service-learning programs enhances connectivity, reduces the risk of losing the motivation to maintain an active and healthy lifestyle [8,25], and improves quality of life [26]. It also gives the older adults the opportunity to challenge stereotypes and experience a feeling of successful aging [25]. Second, among college students, (younger generation), intergenerational service-learning improves their academic and professional learning skills, as well as increase their social awareness with respect to the struggles faced by disadvantaged communities, such as ageism [25,27].

### 1.2. Hypotheses

Based on the above premises, it is hypothesized that:

**Hypothesis** **1.** *Compared with non-service-learning students, service-learning students will exhibit a lower level of ageism after service-learning completion.*

**Hypothesis** **2.** *Compared with non-service-learning students, service-learning students will exhibit a higher level of competency and comfort interacting with senior individuals.*

**Hypothesis** **3.** *Senior individuals will exhibit a lower level of ageism after service-learning completion.*

**Hypothesis** **4.** *Senior individuals will exhibit a lower level of loneliness after service-learning completion.*

## 2. Materials and Methods

### 2.1. Study Design

This project was designed in the middle of the Novel Coronavirus (COVID-19) pandemic, during the fall 2020 semester. Therefore, one very important question centers on whether “virtual” interactions between seniors and students can have a positive impact on students’ perceptions of seniors (the positivity or negativity of their attitudes toward seniors) and seniors’ perceptions of younger persons (again measured as positive or negative attitudes), and senior feelings of social competency and loneliness. During the prior Spring 2020 semester, a service-learning project placed students and seniors together for weekly interactions, but the number of those interactions was cut short (from eight weeks to four weeks) because of the Pandemic. Nonetheless, the researchers were able to show that limited interactions between students and seniors significantly decreased students’ scores on a measure of ageism, significantly increased student ratings of the degree to which they Know, Care, and Act on multicultural issues, and significantly increased student self-ratings of confidence in interacting with individuals of an older generation [27].

The occurrence of the pandemic and the subsequent need to switch to a 100% virtual service-learning method led the current researchers to consider whether virtual interactions can have as a positive impact as those demonstrated in previous semesters. In a 2020 meta-analysis, Ibarra and colleagues summarized findings on the possible impact of use of technology on senior feelings of social disconnectedness and social isolation. Though most results across studies were modest, the researchers concluded that technology could be effective in reducing seniors’ feelings of social disconnectedness and social isolation, but studies assessing that impact needed to have more rigorous methods employed and that technologies used should also be assessed [28]. The current study attempted to assess the impact of “virtual” interactions between undergraduate college students and seniors, using an experimental and control group design, on senior feelings of loneliness, social competency, and attitudes toward a younger adult, as well as the impact on younger adults’ attitudes toward seniors.

### 2.2. Study Participants

During the fall 2020 semester, undergraduate students enrolled in a course provided by the School of Health Administration, with a special emphasis on cultural competency and diversity, participated in this study. A group of students were randomly assigned to the “service-learning” project (*n* = 18). The project consisted of pairing students with seniors living in the communities surrounding the university. Twenty-two seniors participated in this service-learning project. Students were required to have at least a 30-min weekly virtual interaction with a senior individual either via phone, text messages, or face-to-face on Zoom or another platform, for six weeks. Students were expected to write a half-page diary on each interaction. In addition, students were asked to write a one-page reflection after three weeks of interaction, for a total of two reflections.

Students not assigned to the service-learning project (*n* = 24) were required to write a five-page academic paper on ageism’s manifestation in several countries. The instructor provided non-service-learning students with the outline of the ageism paper. Both student diaries and reflections as well as ageism papers were graded at the same scale and grades were included in students’ final semester grades.

### 2.3. Tools for Data Collection

To measure service-learning effectiveness, students were asked to fill out pre- and post-surveys administered on Qualtrics. In addition, to measure the effectiveness of intergenerational service-learning on loneliness, senior partners were expected to fill out a pre-post survey (paper-based survey). This study was approved by the Institutional Review Board of the authors’ institution (IRB # 7046). Written informed consents were issued to both students and seniors when the surveys were deployed.

### 2.4. Study Procedure

Three measures were employed in a pre-post method in this study. The Aging Semantic Differential (ASD) Scale, administered to both students and seniors, [29,30,31], the UCLA Loneliness Scale, administered to seniors [32], a one-item researcher generated Likert-rating of loneliness on a 10-point scale, administered to the seniors (1 = not at all lonely to 10 = very lonely), and two-item researcher generated Likert-rating of student competency interacting with senior individuals. Competency was measured in terms of comfort and confidence levels interacting with individuals of older generation (1 = not at all comfortable and/or not at all confident to 10 = very comfortable and/or very confident). The ASD scale has been found to have high levels of reliability and validity, at least in studies in applied program. Reliability and validity computed in a project with nursing students, for example, comparing scores on ASD Scale in comparison to Big 5 Personality dimensions and Positive and Negative Affect Scale. Results showed the ASD scale to have a Cronbach alpha of 0.92 for seniors and 0.93 for young persons in comparison to the PANAS and roughly 0.75 for younger participants and 0.83 for older participants. This differential scale provides attitudinal dimensions (such as Progressive 1-----2-----3-----4-----5-----6-----7 Old Fashioned) that are considered to be opposites [30,31].

Researchers have found the UCLA Loneliness Scale to be less amenable to reliable and valid analyses than other scales, predominantly because of a lack of a simple and reliable assessment technique. The development of the UCLA Loneliness Scale, a short, 20-item general measure of loneliness is reported. The measure has high internal consistency (coefficient alpha = 0.96) and a test-retest correlation over a two-month period of 0.73. Concurrent and preliminary construct validity are indicated by correlations with self-reports of current loneliness and related emotional states, and by volunteering for a “loneliness clinic.” [32].

### 2.5. Data Analysis

Sample *t*-tests were used for our pre-post design. Data cleaning and analysis were conducted using IBM-SPSS version 26. The survey instruments used in this study are presented in the Supplementary Materials section.

## 3. Results

### 3.1. Student Assessment Results

All measures for students (ASD Scale, Ageism, and Competency to Work with Older Adults) were administered both pre- and post-interactions with seniors. Additionally, some students (*n* = 18) interacted with seniors while the remaining students (*n* = 24) completed a more traditional research project on ageism rather than interact with the seniors. The basic results for both the pre- and the post-measures for the service and non-service-learning students are summarized in Table 1.

As can be seen in Table 1, the two groups do not differ significantly on any of the variables at the beginning of the project (Pretest data), but the service-learning group shows significantly lower ASD scores (more positive semantic differential ratings) at the posttest, in comparison to the non-service-learning students, t (1, 40) = −2.027, *p* = 0.049. Additionally, the service-learning group has significantly lower ageism scores on the posttest, in comparison to the non-service-learning group, t (1, 40) = −2.102, *p* = 0.042. This means the service-learning group ageism scores have dropped from pretest to posttest while the non-service-learning group scores have remained relatively the same. Therefore, *Hypothesis 1* is supported.

Lastly, the change in competency scores on the posttest, comparing the service-learning to non-service-learning groups, is not significant—though the service-learning group is rating themselves as more competent working with seniors at the end of the project than at the beginning, but so are the non-service-learning group t (1, 40) = 1.544, *p* = 0.13). Therefore, *Hypothesis 2* is not supported.

### 3.2. Senior Assessment Results

Twenty-two seniors engaged in virtual interactions with the students for this project for at least 30 min per week for six weeks. As a reminder, seniors completed three measures both before and after the six weeks of interactions with students. Seniors were given the ASD Scale (asking them to rate their attitudes differentially toward younger persons), the UCLA Loneliness Scale, and a researcher generated question assessing loneliness on a 10-point Likert scale. Analyses were conducted on twenty seniors as two did not complete both pre- and post-measures.

Scores on the differential scale (with higher scores reflecting more negative choices on the differentials and higher ratings) have decreased from pre to post assessment but the decrease has not reached significance (Means of 100.65 and 90.50 at pre and post, respectively), t (1, 19) = 1.483, *p* = 0.154. This means the seniors are rating younger persons more positively on the differentials at the end of the six weeks of interaction than they were at the beginning of the project, but the change is not large enough to reach significance. Therefore, *Hypothesis 3* is not supported.

Seniors were also given the UCLA Loneliness Scale. As a reminder to the reader, this is a 20-item measure that provides statements about loneliness (such as “I lack companionship” or “I feel completely alone”) and provides a 4-response rating scale for the frequency of those feelings (0 = often, S = sometimes, R = rarely and N = Never). As predicted, scores on the UCLA Scale (with higher numbers reflecting more loneliness) have dropped significantly from pre- to post-interactions with students (Means of 73.65 and 68.30 for pre- and post-intervention, respectively), t (1, 19) = 2.301, *p* = 0.033 (See Table 2)

As predicted, scores on the one-item rating scale of loneliness (with higher scores reflecting more loneliness) decreased significantly from pre to post assessment (Means of 3.80 and 2.40 at pre and post, respectively), t (1, 22) = 2.412, *p* = 0.009. This means the seniors are rating themselves as less lonely at the end of the six weeks of virtual interaction than they were at the beginning of the project (See Table 2). Therefore, *Hypothesis 4* is supported.

## 4. Discussion

By engaging undergraduate college students in a virtual service-learning project with the aim to reduce perceived loneliness among individuals of older generation, increase college students’ competenciesinteracting with senior individuals, and reduce mutual age-related bias toward the older and younger generations, this study found that service-learning was effective in achieving these aims. The results of this study highlight the success of service-learning, even at a virtual setting, in reducing age-related bias, among college students, and perceived loneliness, among the elderly.

While we did not find a significant difference in students’ competency and comfort interacting with individuals of older generation by comparing service-learning with non-service-learning students using the post-intervention survey data, we found that service-learning students exhibited a greater mean difference between pre- and post-intervention scores (16.83 vs. 19.72), compared with non-service-learning students (17.54 vs. 18.25) (See Table 1). This finding suggests that writing a five-page paper on ageism may have increased students’ awareness of the issue as well as their understanding of seniors, but it is not as effective as having a virtual interaction with senior partners and writing a half-page diary on each interaction, as well as writing a whole-page reflection after three interactions. Based on this finding, educators in several settings and different countries can implement this effective and efficient intergenerational service-learning pedagogy to reduce ageism among college students.

With respect to the effect of the service-learning intervention on ageism and loneliness among senior partners, the intervention is not significantly effective in reducing bias toward the younger generation, as measured by the ASD scale, but it is significantly effective in reducing loneliness level. Therefore, engaging in a weekly virtual interaction with senior individuals is a highly cost-effective intervention to alleviate the burden of loneliness among this population. As such, since loneliness is a global pandemic, this kind of intergenerational service-learning that can be implemented to reduce loneliness worldwide.

### Limitations

This study has some limitations. First, while the original plan was for students to virtually engage in the service-learning project for eight weeks, delay in IRB approval prevented us from starting the project on time, therefore cutting the number of weeks from eight to six. Regardless, we found that six weeks were enough to achieve our aims. Second, the findings from this study are not generalizable because we used a sample of students from a particular course. However, the study design is replicable in other service-learning settings. Third, given the short-term span of the project, as well as the absence of clinician students, our study did not cover the impact of intergenerational service-learning on the clinical outcomes of senior participants, such as blood pressure level, blood sugar level, weight, happiness and stress hormones, and longevity.

Our study is also limited with respect to small sample size. Consequently, we were not able to conduct regression analyses to control for other factors that may have influenced ageism and loneliness. However, *t*-tests are the recommended data analysis tools for pre-post design when the sample size is small.

While this study is purely quantitative, students’ diaries and reflections also contain a wealth of information regarding their feelings throughout the project. A qualitative study analyzing student feelings could also be an additional means to assess the effectiveness of this service-learning intervention.

## 5. Conclusions

The purpose of this study was to assess the effectiveness of intergenerational service-learning on ageism, among undergraduate college students, and its effect on ageism and loneliness, among senior individuals. Students were paired with senior individuals to have at least a 30-min virtual conversation for six weeks. We tested four hypotheses. This study demonstrated that the designed virtual intergenerational service-learning reduced ageism bias, among college students (Hypothesis 1) and mitigated perceived loneliness among senior individuals (Hypothesis 4).

The reduction in ageism bias, among college students, after the completion of the intergenerational service-learning, was consistent with the findings from prior studies [27,33]. While these prior studies designed service-learning as a face-to-face interaction between college students and seniors, the service-learning of this current study was conducted on a virtual platform. Additionally, a meta-analysis assessing the effectiveness of various interventions on ageism attitudes against older adults indicated that intergenerational contact significantly decreases ageism bias among students [34].

With respect to the impact of our study on the older generation, our study did not significantly affect seniors’ ageism bias toward the younger generation. The ageism score level decreased at the completion of the project, but the decrease was not statistically significant. However, our study significantly reduced the level of loneliness among the seniors. That finding is contrary to Shaw’s finding that intergenerational program did not decrease the sense of loneliness among seniors [35]. The discrepancy in the findings between our study and Shaw’s could be explained by the difference in study design and analysis. We used a pre-post design while Shaw used correlation.

Given that the COVID-19 pandemic exacerbated the loneliness level of everyone and especially senior individuals, this kind of pandemic should not deter us from capitalizing on technology and engaging senior individuals in virtual interactions to alleviate loneliness burden. While the cost of such interaction is minimal, the gain can be substantial given the negative effects of loneliness on health outcomes. Service-learning is a two edged-sword that can both solve a social issue and train students in social engagement [36]. Therefore, college programs with a special focus on social issues could incorporate service-learning in their curriculum as it has proven to be an effective and efficient means to both educate students and solve social issues, such as hunger, racism and discrimination, ageism, poverty, homelessness, physical disability, education, and legislative advocacy [36].

This study contributes to the field of intergenerational service-leaning study, more specifically in addressing ageism (among college students) and loneliness (among senior adults) in a virtual setting. This could serve as a model that educators worldwide can adopt, expand on, or improve.

## Figures and Tables

**Table 1:** Pre-Post Student Assessment Results.

	Group ^a^	N	Mean	Std. Deviation	Std. Error Mean
ASD ^b^	1.00	18	109.5556	16.49322	3.88749
	2.00	24	116.4583	17.32798	3.53706
Ageism	1.00	18	46.5000	8.78669	2.07104
	2.00	24	43.5833	10.34583	2.11183
Competency	1.00	18	16.8333	4.27372	1.00733
	2.00	24	17.5417	3.94505	0.80528
ASDPost *	1.00	18	95.6111	18.17930	4.28490
	2.00	24	109.7917	25.12227	5.12806
AgeismPost *	1.00	18	35.1667	12.21980	2.88023
	2.00	24	42.9167	11.52659	2.35286
CompetencyPost	1.00	18	19.722	1.96456	0.46305
	2.00	24	18.250	3.66238	0.74758

^a^ Group 1—SL Group, Group 2-Non-SL Group; ^b^ Aging Semantic Differential Scale. * Mean difference of the two groups significant at *p* ≤ 0.05.

**Table 2:** Pre-Post Senior Assessment Results.

	Group ^a^	N	Mean	Std. Deviation	Std. Error Mean
ASD ^b^	1.00	22	100.65	20.88131	4.66920
ASD Post			90.50	21.35046	4.77411
UCLA Loneliness Scale	1.00	22	73.65	5.59393	1.25084
UCLA Loneliness ScalePost ^!^			68.30	9.40940	2.10401
One-item Loneliness Scale	1.00	22	3.80	2.191	0.490
One-item Loneliness ScalePost ^!!^			2.412	1.31389	0.29330

^a^ One group of senior adults; ^b^ Aging Semantic Differential Scale; ^!^ Mean difference pre and post of the same group significant at *p* ≤ 0.05. ^!!^ Mean difference pre and post of the same group significant at *p* ≤ 0.01.

## Data Availability

The IRB of this study does not allow data sharing because data protection statement is an integral part of our IRB approval. Therefore, our data will not be made available.

## Supplementary Materials

The following supporting information can be downloaded at: https://www.mdpi.com/article/10.3390/healthcare10050893/s1, Survey Instruments.
