# Supplementary material for: Assessing the Effectiveness of Intergenerational Virtual Service-Learning Intervention on Loneliness and Ageism: A Pre-Post Study
Source: Healthcare (Basel). 2022 May 12;10(5):893. doi: 10.3390/healthcare10050893 (PMC9141066; doi:10.3390/healthcare10050893)
Supplement: Supplementary file 1 [file healthcare-10-00893-s001.zip › healthcare-1687515-supplementary.pdf]

Aging Semantic Differential with 7-Point Likert Scale [30, 31]

Assessing Senior Attitudes Toward Younger Individuals (undergraduate students)

Please rate the following statements on a scale of 1 to 7.

Most younger adults are:

Progressive 1------ --2----------3------ ----4----------5----------6-----------7 Old-fashioned

Consistent 1---------2----------3---------4----------5----------6-----------7 Inconsistent

Independent 1---------2----------3---------4----------5----------6-----------7 Dependent

Rich 1---------2----------3---------4----------5----------6-----------7 Poor

Generous 1---------2----------3---------4----------5----------6-----------7 Selfish

Productive 1---------2----------3---------4----------5----------6-----------7 Unproductive

Busy 1---------2----------3---------4----------5----------6-----------7 Idle

Secure 1---------2----------3---------4----------5----------6-----------7 Insecure

Strong 1---------2----------3---------4----------5----------6-----------7 Weak

Healthy 1---------2----------3---------4----------5----------6-----------7 Unhealthy

Active 1---------2----------3---------4----------5----------6-----------7 Passive

Handsome 1---------2----------3---------4----------5----------6-----------7 Ugly

Cooperative 1---------2----------3---------4----------5----------6-----------7 Uncooperative

Optimistic 1---------2----------3---------4----------5----------6-----------7 Pessimistic

Satisfied 1---------2----------3---------4----------5----------6-----------7 Dissatisfied

Expectant 1---------2----------3---------4----------5----------6-----------7 Resigned

Flexible 1---------2----------3---------4----------5----------6-----------7 Inflexible

Hopeful 1---------2----------3---------4----------5----------6-----------7 Dejected

Organized 1---------2----------3---------4----------5----------6-----------7 Disorganized

Happy 1---------2----------3---------4----------5----------6-----------7 Sad

Friendly 1---------2----------3---------4----------5----------6-----------7 Unfriendly

Neat 1---------2----------3---------4----------5----------6-----------7 Untidy

Trustful 1---------2----------3---------4----------5----------6-----------7 Suspicious

Self-reliant 1----------2-----------3----------4-----------5-----------6------------7 Dependent

Liberal 1----------2-----------3----------4-----------5-----------6------------7 Conservative

Certain 1----------2-----------3----------4-----------5-----------6------------7 Uncertain

Tolerant 1----------2-----------3----------4-----------5-----------6------------7 Intolerant

Pleasant 1----------2-----------3----------4-----------5-----------6------------7 Unpleasant

Ordinary 1----------2-----------3----------4-----------5-----------6------------7 Eccentric

Aggressive 1----------2-----------3----------4-----------5-----------6------------7 Defensive

Exciting 1----------2-----------3----------4-----------5-----------6------------7 Dull

Decisive 1----------2-----------3----------4-----------5-----------6------------7 Indecisive

Semantic Differential with 7-point Likert Scale [30, 31]
Assessing Younger Individuals' (undergraduate students) Attitudes Toward Seniors

Please rate the following statements on a scale of 1 to 7.

Most senior adults are:

Progressive 1----------2-----------3----------4-----------5-----------6------------7 Old-fashioned

Consistent 1----------2-----------3----------4-----------5-----------6------------7 Inconsistent

Independent 1----------2-----------3----------4-----------5-----------6------------7 Dependent

Rich 1----------2-----------3----------4-----------5-----------6------------7 Poor

Generous 1----------2-----------3----------4-----------5-----------6------------7 Selfish

Productive 1----------2-----------3----------4-----------5-----------6------------7 Unproductive

Busy 1----------2-----------3----------4-----------5-----------6------------7 Idle

Secure 1----------2-----------3----------4-----------5-----------6------------7 Insecure

Strong 1----------2-----------3----------4-----------5-----------6------------7 Weak

Healthy 1----------2-----------3----------4-----------5-----------6------------7 Unhealthy

Active 1----------2-----------3----------4-----------5-----------6------------7 Passive

Handsome 1----------2-----------3----------4-----------5-----------6------------7 Ugly

Cooperative 1----------2-----------3----------4-----------5-----------6------------7 Uncooperative

Optimistic 1----------2-----------3----------4-----------5-----------6------------7 Pessimistic

Satisfied 1----------2-----------3----------4-----------5-----------6------------7 Dissatisfied

Expectant 1----------2-----------3----------4-----------5-----------6------------7 Resigned

Flexible 1----------2-----------3----------4-----------5-----------6------------7 Inflexible

Hopeful 1----------2-----------3----------4-----------5-----------6------------7 Dejected

Organized 1----------2-----------3----------4-----------5-----------6------------7 Disorganized

Happy 1----------2-----------3----------4-----------5-----------6------------7 Sad

Friendly 1----------2-----------3----------4-----------5-----------6------------7 Unfriendly

Neat 1----------2-----------3----------4-----------5-----------6------------7 Untidy

Trustful 1----------2-----------3----------4-----------5-----------6------------7 Suspicious

Self-reliant 1----------2-----------3----------4-----------5-----------6------------7 Dependent

Liberal 1----------2-----------3----------4-----------5-----------6------------7 Conservative

Certain 1----------2-----------3----------4-----------5-----------6------------7 Uncertain

Tolerant 1----------2-----------3----------4-----------5-----------6------------7 Intolerant

Pleasant 1----------2-----------3----------4-----------5-----------6------------7 Unpleasant

Ordinary 1----------2-----------3----------4-----------5-----------6------------7 Eccentric

Aggressive 1----------2-----------3----------4-----------5-----------6------------7 Defensive

Exciting 1----------2-----------3----------4-----------5-----------6------------7 Dull

Decisive 1----------2-----------3----------4-----------5-----------6------------7 Indecisive

The UCLA Loneliness Scale (Senior Survey) [32]

Indicate how often each of the statements below is descriptive of you. Circle one letter for each statement:

0 indicates "I often feel this way"

S indicates "I sometimes feel this way"

R indicates "I rarely feel this way"

N indicates "I never feel this way".

1. I am unhappy doing so many things alone. .... 0 S R N

2. I have nobody to talk to. .... 0 S R N

3. I cannot tolerate being so alone. .... 0 S R N

4. I lack companionship. .... 0 S R N

5. I feel as if nobody really understands me. .... 0 S R N

6. I find myself waiting for people to call or write. .... 0 S R N

7. There is no one I can turn to. .... …0 S R N

8. I am no longer close to anyone. .... 0 S R N

9. My interests and ideas are not shared by those around me. .... 0 S R N

10. 1 feel left out. .... 0 S R N

11. I feel completely alone. ....….0 S R N

1 2. I am unable to reach out and communicate with those around me…..….0 S R N

13. My social relationships are superficial. .... 0 S R N

14. I feel starved for company. .... 0 S R N

15. No one really knows me well. .... 0 S R N

16. I feel isolated from others. .... 0 S R N

17. I am unhappy being so withdrawn. .... 0 S R N

18. It is difficult for me to make friends. .... 0 S R N

19. I feel shut out and excluded by others. .... 0 S R N

20. People are around me but not with me. .... 0 S R N

Rate from a scale of 1 to 10 your level of loneliness. **1 stands for "I do not feel lonely"** and **10 stands for "I feel very lonely".**

1(I do not feel lonely) --**10** (I feel very lonely)

Ageism Scale (Student survey) [29]

Please rate the following statements on a scale of 1 (strongly agree) to 7 (strongly disagree)

1	2	3	4	5	6	7
strongly disagree	disagree moderately	disagree slightly	neutral	agree slightly	agree moderately	agree strongly

1. It is good to tell old people that they are too old to do certain things; otherwise, they might get their feelings hurt when they eventually fail.

2. Even if they want to, old people shouldn't be allowed to work because they have already paid their debt to society.

3. Even if they want to, old people shouldn't be allowed to work because they are fragile and may get sick.

4. It is good to speak slowly to old people because it may take them a while to understand things that are said to them.

5. People should shield older adults from sad news because they are easily moved to tears.

6. Older people need to be protected from the harsh realities of society.

7. It is helpful to repeat things to old people because they rarely understand the first time.

8. Even though they do not ask for help, older people should always be offered help.

9. Even if they do not ask for help, old people should be helped with their groceries.

10. Most old people interpret innocent remarks or acts as being ageist.

11. Old people are too easily offended.

12. Old people exaggerate the problems they have at work.

13. Old people are a drain on the health care system and the economy.

Multicultural Sensitivity Scale (Student Survey)

Rate from a scale of 1 to 10 your comfort in interacting with individuals of older generation.
Rate from a scale of 1 to 10 your confidence in interacting with individuals of older generation.
